# Improvement of vertebral body fracture reduction utilizing a posterior reduction tool: a single-center experience

**DOI:** 10.1186/s13018-023-03793-7

**Published:** 2023-04-25

**Authors:** Martin F. Hoffmann, Kristina Kuhlmann, Thomas A. Schildhauer, Katharina E. Wenning

**Affiliations:** Berufsgenossenschaftliches Universitätsklinikum Bergmannsheil Bochum, Bürkle-de-La-Camp-Platz 1, 44789 Bochum, Germany

**Keywords:** Vertebral fracture, Thoracolumbar spine, Posterior surgical treatment, Reduction tool

## Abstract

**Background:**

Extensive research regarding instabilities and prevention of kyphotic malalignment in the thoracolumbar spine exists. Keystones of this treatment are posterior instrumentation and anterior vertebral height restoration. Anterior column reduction via a single-stage procedure seems to be advantageous regarding complication, blood loss, and OR-time. Mechanical elevation of the anterior cortex of the vertebra may prevent the necessity of additional anterior stabilization or vertebral body replacement. The purpose of this study was to examine (1) if increased bony reduction in the anterior vertebral cortex could be achieved by utilization of an additional reduction tool, (2) if postoperative loss of vertebral height could be reduced, and (3) if anterior column reduction is related to clinical outcome.

**Methods:**

From one level I trauma center, 173 patients underwent posterior stabilization for fractures of the thoracolumbar region between 2015 and 2020. Reduction in the vertebral body was performed via intraoperative lordotic positioning or by utilization of an additional reduction tool (Nforce, Medtronic). The reduction tool was mounted onto the pedicle screws and removed after tightening of the locking screws. To assess bony reduction, the sagittal index (SI) and vertebral kyphosis angle (VKA) were measured on X-rays and CT images at different time points ((1) preoperative, (2) postoperative, (3) ≥ 3 months postoperative). Clinical outcome was assessed utilizing the Ostwestry Disability Index (ODI).

**Results:**

Bisegmental stabilization of AO/OTA type A3/A4 vertebral fractures was performed in 77 patients. Thereof, reduction was performed in 44 patients (females 34%) via intraoperative positioning alone (control group), whereas 33 patients (females 33%) underwent additional reduction utilizing a mechanical reduction tool (instrumentation group). Mean age was 41 ± 13 years in the instrumentation group (IG) and 52 ± 12 years in the control group (CG) (*p* < 0.001). No differences in terms of gender and comorbidities were found between the two groups. Preoperatively, the sagittal index (SI) was 0.69 in IG compared to 0.74 in CG (*p* = 0.039), resulting in a vertebral kyphosis angle (VKA) of 15.0° vs. 11.7° (*p* = 0.004). Intraoperatively, a significantly greater correction of the kyphotic deformity was achieved in the IG (*p* < 0.001), resulting in a compensation of the initially more severe kyphotic malalignment. The SI was corrected by 0.20–0.88 postoperatively, resulting in an improvement of the VKA by 8.7°–6.3°. In the CG, the SI could be corrected by 0.12–0.86 and the VKA by 5.1°–6.6°. The amount of correction was influenced by the initial deformity (*p* < 0.001). Postoperatively, both groups showed a loss of correction, resulting in a gain of 0.08 for the SI and 4.1° in IG and 0.03 and 2.0°, respectively. The best results were observed in younger patients with initially severe kyphotic deformity. Considering various influencing factors, clinical outcome determined by the ODI showed no significant differences between both groups.

**Conclusion:**

Utilization of the investigated reduction tool during posterior stabilization of vertebral body fractures in a suitable collective of young patients with good bone quality and severe fracture deformity may lead to better reduction in the ventral column of the fractured vertebral body and angle correction. Therefore, additional anterior stabilization or vertebral body replacement may be prevented.

## Background

The thoracolumbar spine represents the central static and dynamic structural element of the human body and accounts for approximately 35% of body size in adult humans. Fractures in the region of the thoracic and lumbar spine are common entities representing 80% of all vertebral fractures [1]. Almost half (49.6%) of vertebral body fractures are reported to be burst or incomplete burst fractures [2, 3]. Thereof, the majority (68%) of these fractures occur at the thoracolumbar junction (Th11-L2) [2]. Despite more than 50 years of experience and extensive research, no consensus exists about preferable techniques and approaches. There is a permanent confusion regarding the best treatment for fractures of the thoracolumbar junction with an increasing body of evidence suggesting that these fractures are best treated by a combined posterior and anterior approach [4–11].

Anatomic fracture reduction in thoracolumbar fractures is essential in restoring the alignment in this region. A wide variety of instrumentation systems and procedures is available for the treatment of vertebral body fractures in the thoracolumbar junction. For most techniques, surgical goals are restoration of sagittal alignment via reduction in the anterior vertebral cortex and maintenance of a stable fixation to permit early mobilization [12]. Posterior stabilization offers a great potential for reduction [2, 8]. Additionally, for spine surgeons, the posterior approach is often more familiar and with lower morbidity [13–16]. Therefore, an initial posterior stabilization is performed in the majority of patients treated surgically [2]. Recently, a novel reduction tool (NForce®, Medtronic, Minneapolis, MN) has been introduced to improve reduction in spinal fractures during open or minimal invasive posterior stabilization with the Longitude®/Solera® system (Medtronic, Minneapolis, MN).

A single previous study could show that the reduction tool was effective in performing percutaneous reduction in spinal fractures, but no comparison was performed [17]. Therefore, the purpose of this study was to examine (1) if increased bony reduction in the anterior vertebral cortex could be achieved by utilization of an additional reduction tool (NForce®, Medtronic, Minneapolis, MN), (2) if postoperative loss of vertebral height could be reduced, and (3) if anterior column reduction is related to clinical outcome.

## Methods

This study was an Institutional Review Board approved retrospective cohort study of consecutive patients undergoing surgical treatment for fractures of the thoracolumbar junction treated with posterior stabilization in a single level I trauma center. During the study period, a total of 593 patients were identified by Current Procedural Terminology (CPT) codes that had initial operative treatment for thoracic and lumbar fractures from January 2015 through June 2020. Inclusion criteria were posterior bisegmental surgical treatment for incomplete and complete burst fractures (AOSpine A3 and A4) of the thoracolumbar junction [18] (Th 11—L2), and age equal to or older than 18 years. Exclusion criteria were additional augmentation, anterior or hybrid stabilization, multiple injuries, metastatic disease or pre-existing infection, and insufficient medical record.

### Surgical treatment

Each patient had two preoperative radiographic views of the thoracolumbar junction. These were an anteroposterior (AP) view and a lateral (lat) view. Each patient also had a CT scan with reconstruction of the spine that provided information on extent of the injury. Furthermore, the CT scan was utilized for classification of the fractures according to the AOSpine classification system [18].

Surgical indications and treatment were performed in accordance with the surgeon’s best knowledge, discretion, and experience. Following the induction of general anesthesia, patients were positioned prone on a radiolucent table with appropriate eye protection and supportive cushions below the chest and pelvis. All patients underwent preoperative reduction by closed reduction maneuvers (positioning, whole-body traction, or manual pressure) [19]. The entire posterior aspect was prepped and draped. A midline incision was performed down to the posterior elements. Attention to detail was maintained to avoid dural or neural element injury. The operative approaches to the spine were tailored to each patient based on the particular location of the fracture and possible soft tissue involvement. No patient underwent spinal decompression or additional spondylodesis. Implants for posterior stabilization (Longitude®/Solera® Sagittal Adjusting Screws, Medtronic, Minneapolis, MN) were inserted through a midline incision utilizing intraoperative standard fluoroscopic ap and lateral views.

**Fig. 1:**
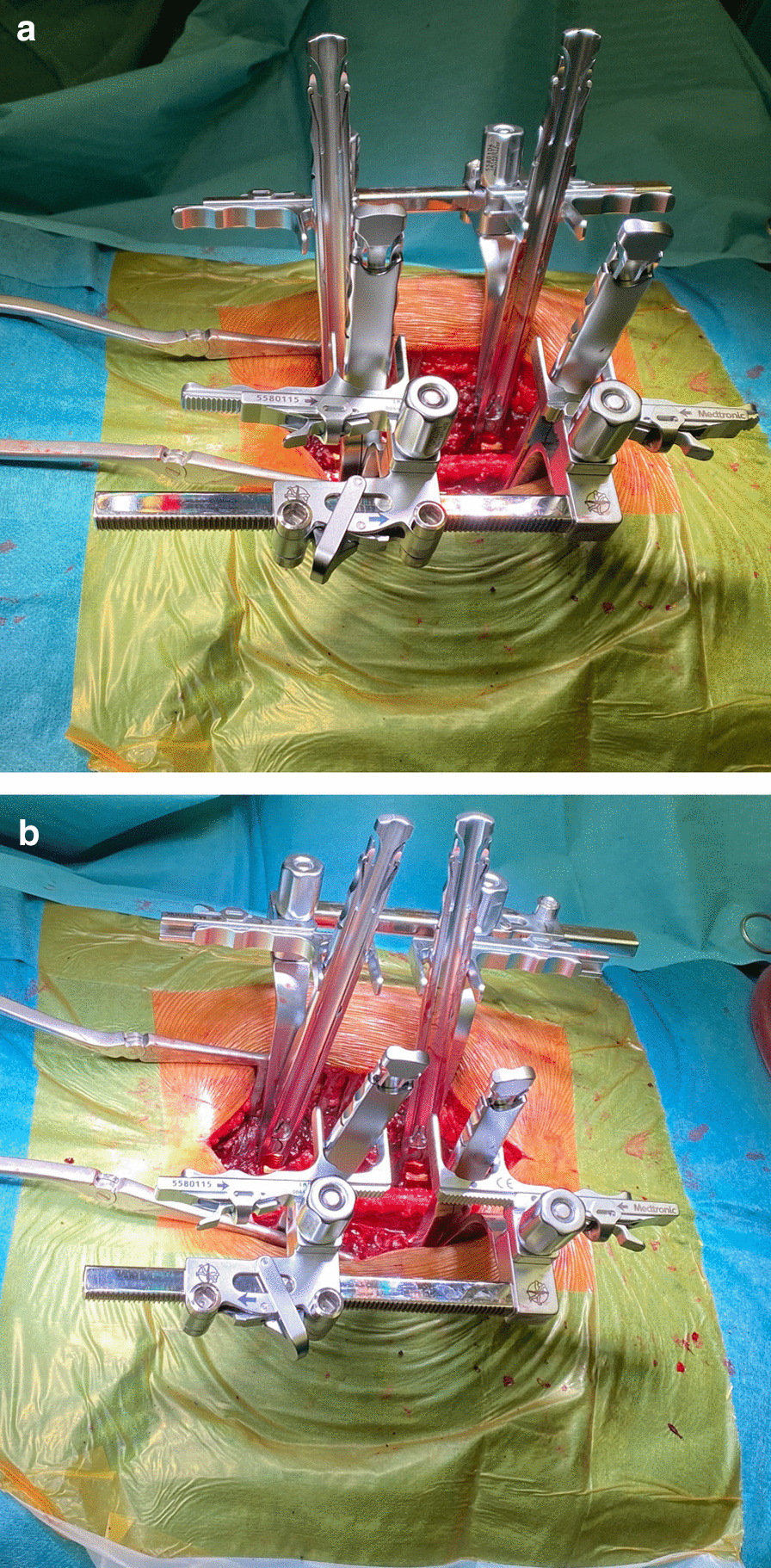
Bilaterally mounted NForce® reduction tool **a** before reduction and **b** after approximation of the lever arms

**Fig. 2:**
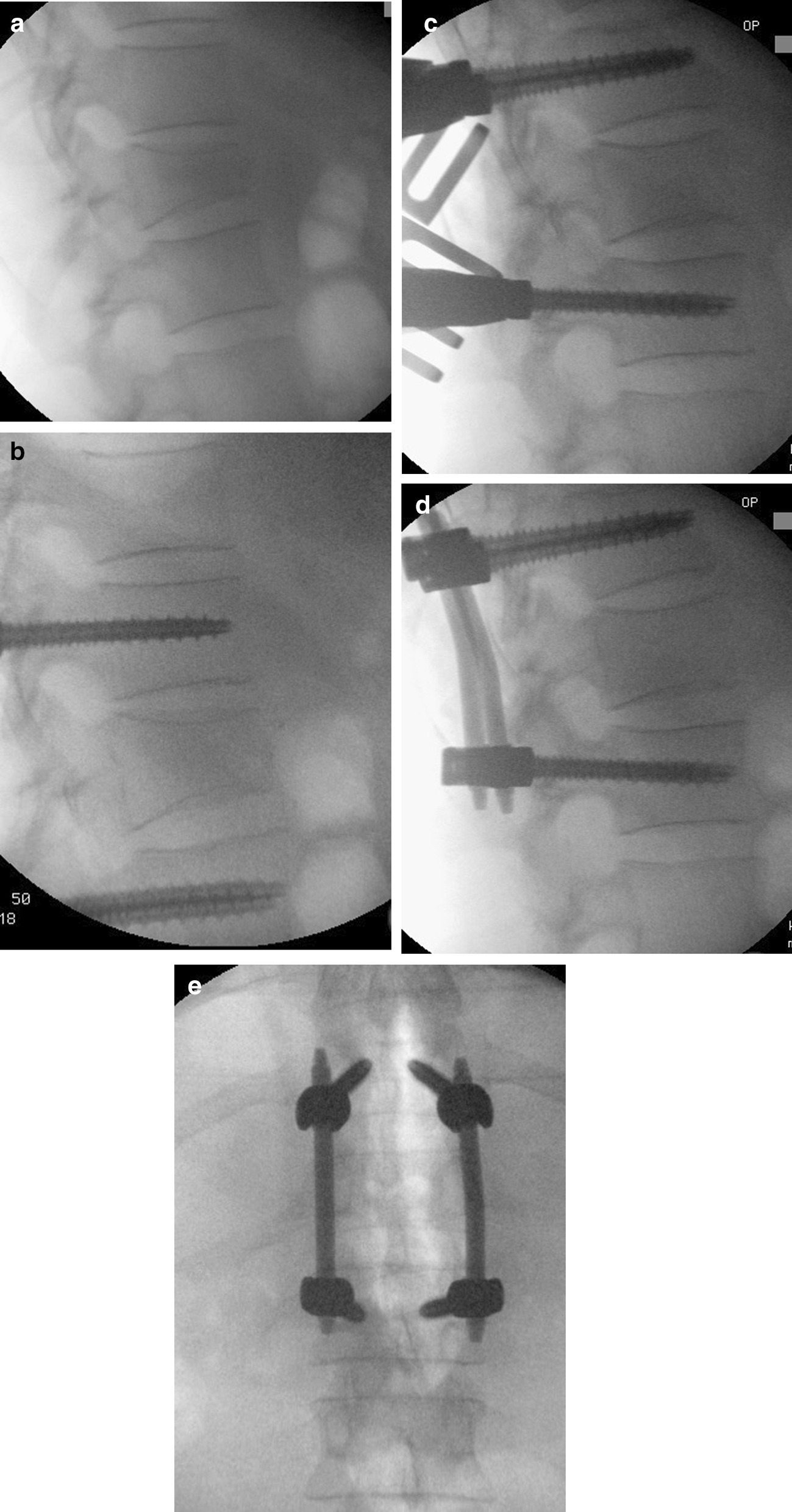
Th12 fracture AOSpine type A3 with kyphotic angulation. **a** intraoperative fluoroscopic view after positioning. **b** vertebral height after positioning of pedicle screws. **c** gain of anterior cortex height via mounted reduction tool. **d** + **e** lateral and ap view of bisegmental posterior instrumentation

For the instrumentation group (IG), reduction in the vertebral body fracture was additionally performed by a reduction device (NForce®, Medtronic, Minneapolis, MN). To perform the reduction, sleeves of the NForce® tool are fixed to the pedicle screws heads. Thereafter, the system needs to be assembled and offers a double-ratchet mechanism (Fig. 1a). Thus, anterior and posterior compression or distraction can be applied separately. Therefore, mounted bilaterally, the system offers the ability to perform multiplanar reduction maneuvers (Fig. 1b). Subsequently, the fractured vertebral body can be gradually reduced via ligamentotaxis by slight distraction of the posterior edge of the vertebral body and anterior spreading by means of lordosis. By proceeding in this kind, a measurable gain in height of the anterior cortex can be achieved. Connecting rods were inserted and fixed by tightening the system (Fig. 2a–e). After thorough irrigation, approaches were closed over drains and in anatomical layers. Skin was closed with vertical Allgower-Donati 3.0 nylon (Ethibond®, Johnson & Johnson, Norderstedt, Germany) sutures. Postoperatively, physiotherapy was initiated immediately without additional stabilization via brace or corset to improve muscle strength and mobility.

### Radiological data assessment

**Fig. 3:**
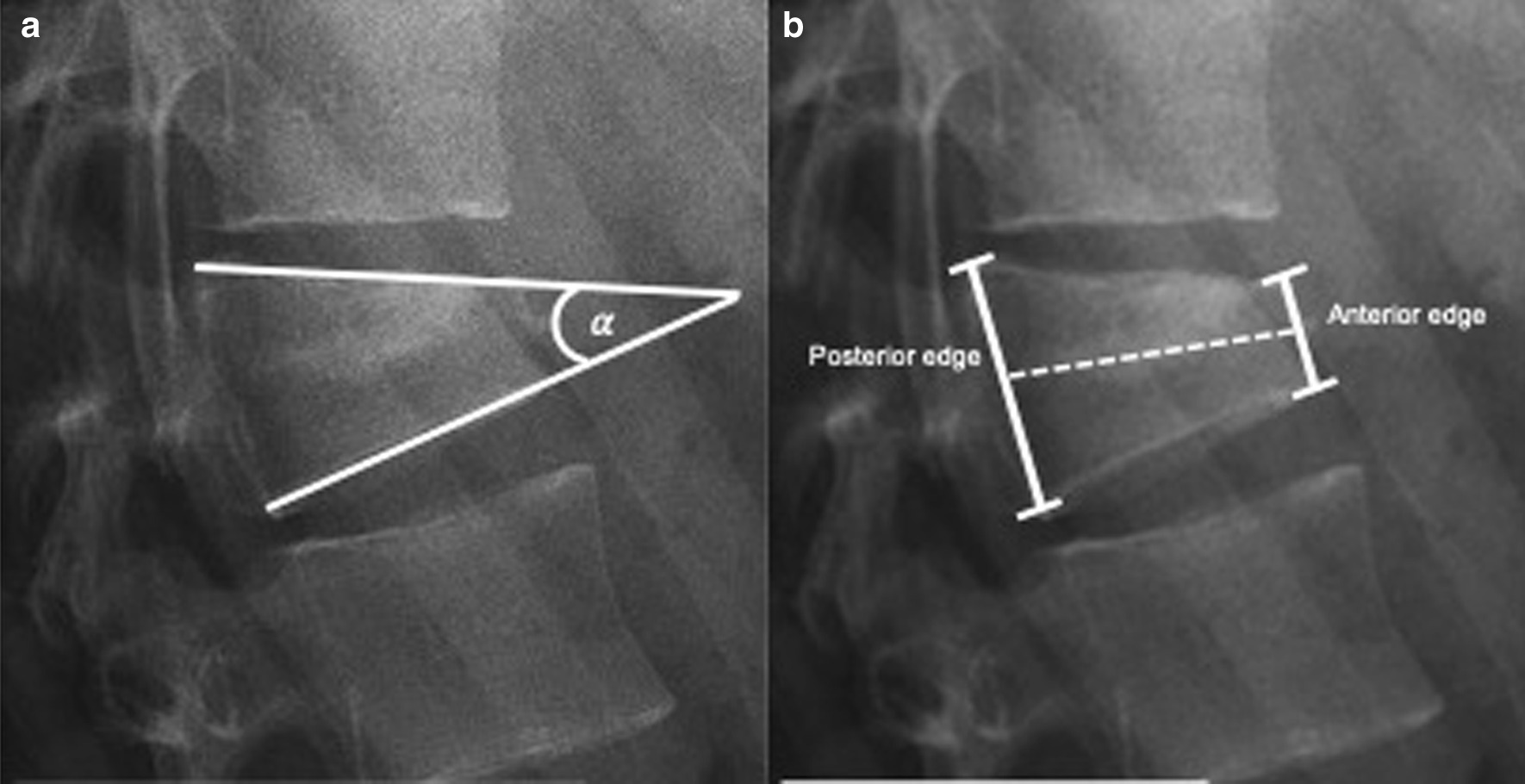
Radiological parameters. **a** vertebral kyphosis angle and **b** sagittal index

All measurements were performed digitally by utilizing the picture archiving and communication system (PACS) (IMPAX 6.6.1, AGFA HealthCare N.V., Belgium). Values were obtained by evaluating X-ray images and CT scans in the lateral view. To assess the bony reduction, sagittal index (SI) (height of the anterior vertebral cortex/height of the posterior vertebral cortex) and vertebral kyphosis angle (VKA) were measured at three different time points [(1) preoperative, (2) immediately postoperative, and (3) after fracture healing ≥ 3 months postoperative] (Fig. 3a and b).

### Clinical data assessment

Injury mechanism, potential contributing factors, and comorbidities were recorded via the hospital documentation program (MEDICO, CGM Europe) (Table 1).

**Table 1:** General patient characteristics of the two study groups at the time of surgery

Parameters		CG (*n* = 44)	IG (*n* = 33)	Statistics
Sex (m/f)		29 (66%)/ 15 (34%)	22 (67%)/ 11 (33%)	${\chi }^{2}$(1) = 0.005, *p* = 0.945, *φ* = 0.01
Age		52 ± 12	41 ± 14	*t*(75) = 3.67, *p* < 0.001
BMI		26 ± 5	26 ± 5	*t*(75) = − 0.15, *p* = 0.885
ASA-Classification	**I** II III	10 (22.7%) 30 (68.2%) 4 (9.1%)	14 (42.4%) 16 (48.5%) 3 (9.1%)	*U* = 596.00, *z* = 1.54, *p* = 0.124
aHT		13 (30%)	9 (27%)	${\chi }^{2}$(1) = 0.05, *p* = 0.827, *φ* = − 0.03
COPD		2 (5%)	0 (0%)	*p* = 0.504, *φ* = − 0.14
Diabetes mellitus		2 (5%)	3 (9%)	*p* = 0.646, *φ* = 0.09

Data with mean and standard deviation ( ±) or absolute numbers and percent. M = male. W = female. BMI = body mass index (kg/m^2^). ASA = American Society of Anesthesiologists. I = normal, healthy patient. II = patient with mild general illness. III = patient with severe general disease. aHT = arterial hypertension. COPD = chronic obstructive pulmonary disease

To evaluate the patient's clinical postoperative satisfaction and health restrictions in daily routine, the Ostwestry Disability Index (ODI) was utilized. The questionnaire was sent to all patients after at least 6 months by mail. The number and quality of returned questionnaires were assessed.

### Statistical analysis

For statistical analysis, Excel (Microsoft® Office Excel©, Version 16.44) and SPSS version 27.0 (IBM, Chicago, IL) were utilized. Continuous data were described by mean (M), median, range (minimum and maximum), and standard deviation (SD). Interval-scaled parameters of the two groups were compared by the T test for independent samples or the Mann–Whitney *U* test. For nominally and ordinally scaled data, the *C*ℎ*i*2 test or *Fischer's exact test* was applied. A two-factorial analysis of variance was performed for the analysis of the radiological data, of the two groups, and comparing the three measurement time points. The correlation between the radiological parameters was tested using Pearson correlation. Changes over each of the two measurement time points (correction, loss, profit) and the questionnaire were evaluated using hierarchical linear regression analysis. A significance threshold of *p* < 0.05 was selected for all statistical tests, and the p-values determined were rounded to three decimal places.

## Results

Seventy-seven (77) patients were included with a mean age of 47 years (range 18–76). There were 51/77 (66%) males and 26/77 (34%) females with a BMI of 26 ± 5 kg/m^2^. Using the AOSpine classification, all fractures were classified as 52 or 53 type A3/A4 fractures.

### Groups

During the study period, 44 patients underwent bisegmental posterior stabilization for fractures of the thoracolumbar junction [18] (Th 11—L2) with preoperative reduction by closed reduction maneuvers (*control group*) and 33 patients underwent the same procedure utilizing the additional reduction device (NForce®, Medtronic, Minneapolis, MN) (*instrumentation group*).

No differences were found between the two groups regarding gender (34% vs. 33% females; *p* = 0.945), BMI (26 ± 5 vs. 26 ± 5 kg/m^2^; *p* = 0.885) or comorbidities. Patients in the control group were older than in the instrumentation group (52 ± 12 years, 41 ± 14 years, respectively; *p* < 0.001) (Table 1).

All patients had a recent history of trauma. Sixty-three of the 77 patients (82%) sustained high-energy trauma. With high-energy fall (61%) being the most common fracture mechanism followed by traffic accident (21%), the most common injury was a fracture of the first lumbar vertebral body (60%) followed by the twelfth thoracic vertebral body (22%). Sixty-four patients had a type A3 fracture, and thirteen patients had a type A4 fracture. There was no significant difference in fracture type or mechanism between the groups (*p* > 0.20) (Table 2).

**Table 2:** Fracture localization and AO type according to groups

		Total	CG (*n* = 44)	IG (*n* = 33)	Statistics
Vertebra	TH11	1% (*n* = 1)	–	3% (*n* = 1)	*p* = 0.250, *φ* = 0.22
	TH12	22% (*n* = 17)	16% (*n* = 7)	30% (*n* = 10)	
	L1	60% (*n* = 46)	66% (*n* = 29)	52% (*n* = 17)	
	L2	17% (*n* = 13)	18% (*n* = 8)	15% (*n* = 5)	
AO-type	A3	83% (*n* = 64)	75% (*n* = 33)	94% (*n* = 31)	${\chi }^{2}$(1) = 4.820, *p* = 0.028, *φ* = − 0.250
	A4	17% (*n* = 13)	25% (*n* = 11)	6% (*n* = 2)	

All data in percent (%) and absolute numbers. Th11 = 11th thoracic vertebra. Th12 = 12th thoracic vertebra. L1 = 1st lumbar vertebra. L2 = 2. lumbar vertebra

### Radiological results

Pre- and postoperative radiographic measurements were available for all patients. Complete radiological data for all three time points were obtained from 56 of the 77 patients (73%), including 28 patients in the CG and 28 patients in the IG. The results at the first two measurement time points did not differ significantly from those of the total collective (*n* = 77). Therefore, only the patients with complete data sets were considered. For the third time point, the mean follow-up period was 14.0 months in the CG (SD = 10.8; median = 12.0; range = 3–43) and 11.0 months in the IG (SD = 7.3; median = 10.5; range = 3–29) (*p* = 0.213) (Table 3).

**Table 3:** Radiological measurements of patients with complete data sets

Radiological Parameters	Measurement time points	CG (*n* = 28) *M* (SD)	IG (*n* = 28) *M* (SD)
SI	1	0.74 (0.11)	0.68 (0.11)
	2	0.87 (0.09)	0.88 (0.10)
	3	0.78 (0.10)	0.76 (0.12)
VKA (°)	1	11.5 (4.5)	15.0 (5.1)
	2	6.1 (3.8)	6.2 (4.7)
	3	9.6 (4.1)	11.1 (4.9)

Mean values (*M*) and standard deviation (SD). SI = sagittal index. VKA = vertebral kyphosis angle

**Fig. 4:**
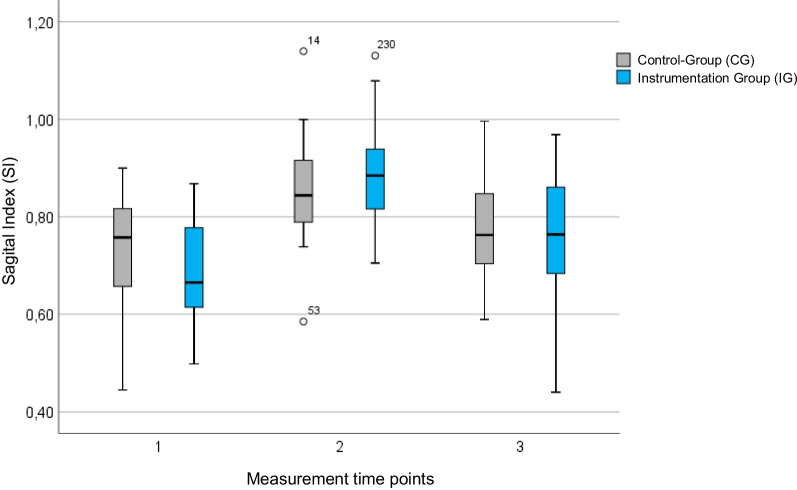
Course of the SI over the three measurement time points. Initially, the SI was corrected by surgery in both groups. Postoperatively, however, there was a loss of correction. In four patients (three of the IG and one of the CG), the anterior cortex was slightly over-distracted compared to the posterior cortex, resulting in an SI > 1

**Fig. 5:**
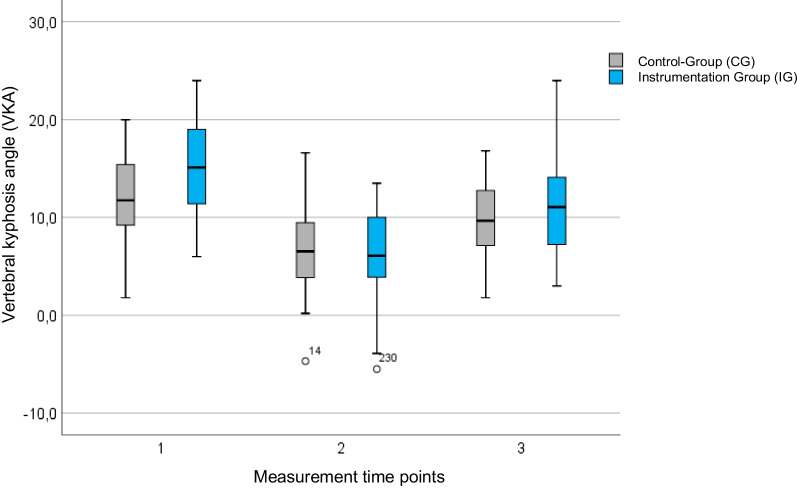
Course of the VKA over the three measurement times. Physiologically, a VKA is around 0°. Positive signs mean a kyphotic position of the vertebra, and negative signs mean a lordotic position of the vertebra

For the SI and VKA, a significant correction (*p* < 0.001) of the initial kyphotic malalignment of the vertebral body could be achieved in both groups (Figs. 4 and 5). Thereof, in four patients (three of the IG and one of the CG), the anterior cortex was slightly over-distracted compared to the posterior cortex, resulting in an SI > 1.

Postoperatively, significant loss of reduction (*p* < 0.001) was observed in both groups. At the third time point, a statistically significant gain for the SI and VKA (*p* < 0.001) compared to the preoperative baseline values was only achieved in the IG. In contrast hereto, there was no significant profit for SI (*p* = 0.201) and VKA (*p* = 0.061) in the control group.

To test the adjusted effect of the reduction tool on the radiographic outcome variables of correction, loss, and profit, a hierarchical linear regression analysis was performed using the independent variables of initial fracture severity, age, sex, and BMI. The analysis could show that the use of a reduction tool had an effect on the extent of correction. Regardless of the initial severity of the kyphosis, age, BMI, and gender, the utilization of the reduction tool resulted in a significantly greater reduction in the fracture compared to the CG (*p* < 0.05).

Additionally, the initial severity of the fracture had a significant influence on the extent of correction. Greater vertebral kyphotic angles resulted in greater correction (*p* < 0.001). On the other hand, patient age had the greatest influence on the postoperative loss of correction (*p* < 0.01). The older the patients and the greater the initial extent of correction, the greater the loss of correction (Table 4). Regarding persistent reduction after bony fracture healing, hierarchical linear regression showed no relevant advantage regarding the utilizations of a reduction tool. Overall young patients with severe initial malalignment had the greatest profit.

**Table 4:** Beta coefficients of hierarchical regression analysis

	Correction SI			Correction VKA		
	*M*1	*M*2	*M*3	*M*1	*M*2	*M*3
Group (0 = CG; 1 = IG)	0.46***	0.32**	0.30*	0.43**	0.25*	0.25*
SI/VKA (1st measuring point)	–	− 0.47***	− 0.52***	–	0.51***	0.58***
Age	–	–	–0.07	–	–	0.02
Sex (0 = male; 1 = female)	–	–	0.14	–	–	0.16
BMI	–	–	–0.07	–	–	− 0.12
*R*-Square	0.21***	0.41***	0.44***	0.19**	0.41***	0.46***

	Loss SI			Loss VKA		
	M1	M2	M3	M1	M2	M3
Group (0 = CG; 1 = IG)	0.22	0.23	0.29*	0.22	0.25	0.33*
SI/VKA (1st measuring point)	–	0.03	− 0.12	–	− 0.07	0.13
Age	–	–	0.29	–	–	0.43**
Sex (0 = male; 1 = female)	–	–	0.12	–	–	0.08
BMI	–	–	0.05	–	–	− 0.02
*R*-Square	0.05	0.05	0.13	0.05	0.05	0.20*

	Profit SI			Profit VKA		
	M1	M2	M3	M1	M2	M3
Group (0 = CG; 1 = IG)	0.24	0.12	0.05	0.24	0.06	− 0.01
SI/VKA (1st measuring point)	–	− 0.43**	− 0.37*	–	0.53***	0.45**
Age	–	–	− 0.26	–	–	− 0.29*
Sex (0 = male; 1 = female)	–	–	0.04	–	–	0.09
BMI	–	–	− 0.10	–	–	− 0.10
*R*-Square	0.06	0.23**	0.29**	0.06	0.30***	0.38***

*p < 0.05, **p < 0.01, ***p < 0.001

Absolute correction/loss/profit as dependent variables; group, SI/KW at 1st measuring point, age, sex, and BMI as independent variables/predictors. SI = sagittal index. VKA = vertebral body angle

M1 = model 1. M2 = model 2. M3 = model 3

### Clinical results

**Fig. 6:**
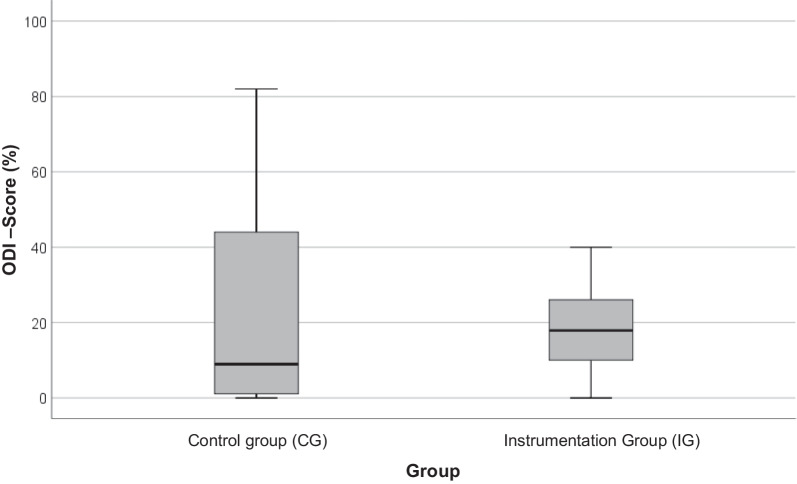
ODI score in percent (%)

The Ostwestry Disability Index (ODI) in its German version was utilized to evaluate clinical outcome after surgical treatment of traumatic vertebral body fracture [20]. The complete ODI was collected from 47 patients. In the CG, the ODI was evaluated in 23 patients (52%) after an average of 34 ± 21 months and in the IG in 24 patients (73%) after an average of 19 ± 10 months (*p* < 0.001). At the time of the ODI survey, patient age averaged 56 ± 13 years in the control group and 42 ± 13 years in the instrumentation group (*p* < 0.001). The average ODI test score in the CG was 21.4% (SD = 23.7; median = 8.9) with a range of 0–82% and thus could be assigned to the category of a “moderate disability.” In the IG, this was 17.7% (SD = 11.8; median = 17.9) with a range of 0–40% and was thus one category below that of the CG (“minimal disability”) (Fig. 6).

To examine factors influencing the ODI test result, again a hierarchical regression analysis was performed. The test result served as the dependent variable and group, time between surgery and ODI collection, SI after bony consolidation, age at the time of ODI collection, hardware removal, and gender as independent variables/predictors. Considering all predictors, patient age and time between surgery and ODI survey had the greatest influence on the ODI test result. None of the selected predictors had a statistically significant impact on patients’ clinical satisfaction (Table 5).

**Table 5:** Beta coefficients of hierarchical regression

Model	ODI-Score (%)			
	*M*1	*M*2	*M*3	*M*4
Group (0 = CG, 1 = IG)	− 0.10	− 0.22	− 0.26	− 0.16
Months between OR and ODI examination	–	− 0.21	− 0.21	− 0.23
SI at third measuring point	–	–	− 0.13	− 0.12
Age	–	–	–	0.28
Sex (0 = male, 1 = female)	–	–	–	0.08
Hardware removal (0 = no, 1 = yes)	–	–	–	− 0.01
*R*-Square	0.01	0.04	0.05	0.13

ODI test score (%) as dependent variable; group, months between OP and ODI survey, SI at third measurement time point, age at ODI survey, and gender as independent variable/predictor. M1 = model 1. M2 = model 2. M3 = model 3. M4 = model 4

## Discussion

Traumatic complete and incomplete fractures of the thoracolumbar junction (type A3/A4 according to the AOSpine classification [18]) are common injuries that usually require surgical intervention [21]. Generally, the potential for reduction seems to be significantly greater with posterior stabilization [2, 8]. Therefore, an initial posterior stabilization is performed in the majority of patients treated surgically [2]. The isolated dorsal procedure is characterized by good initial reduction results and represents a simple procedure with few complications. Furthermore, good functional results are achieved by this procedure, and there is the possibility of a minimally invasive approach [22–24].

Based on radiological results, combined anterior–posterior stabilization seems to improve long-term reconstruction of injured segments [8, 25]. Some authors could not identify clinically a relevant corrective loss after stand-alone posterior instrumentation and highlighted the advantages of this method, such as lower costs and lower morbidity [16, 26, 27]. Additionally, in younger patients that are frequently affected by traumatic vertebral fractures, short-axis instrumentation without fusion can be advantageous by preserving spinal motion segments [28, 29]. Despite the utilization of a reduction tool, among other studies, two multi-center trials initiated by the German Society for Trauma Surgery concluded that the achieved reduction could not be maintained in the long term with solitary posterior instrumentation [30–32]. The reported loss of reduction was based on comparison of the Cobb’s angle, which was postulated to be mainly caused by intervertebral disk space narrowing [5, 10, 33]. Therefore, the focus in our study was exclusively on bony reduction and kyphotic changes of the fractured vertebra. Nevertheless, a comparison of the bony reduction result in isolated dorsal instrumentation depending on the utilization of NForce® for fracture reduction has not yet been performed.

In this study, it was shown that a statistically significant correction of the initial kyphotic malposition of the vertebral body in the context of dorsal instrumentation is possible both by positioning the patient alone and by using a reduction tool. A direct comparison of the groups showed a statistically significant better correction by using the reduction tool. The SI was improved by 0.12 in the control group and by 0.20 in the NForce® group. Consequently, the VKA was improved by 5.1° in the control group and by 8.7° in the instrumentation group. The regression analysis performed showed a significant influence of the radiological baseline on the correction result (p < 0.001). Hence, the more pronounced the deformity of the vertebral body was after the injury, the greater was the correction of the deformity that was surgically achieved.

Additionally, age and BMI seem to be negatively correlated with the extent of correction. Younger patients and patients with lower BMI had a greater potential for correction. However, this influence was not statistically significant in our study. Similar results were reported by Reinhold et al. in a multi-center study [2]. In addition to a significant influence of preoperative Cobb angle on the reduction result, they also observed a relevant contribution of age on the correction.

In conformity with the literature, this study shows a loss of the initially achieved correction at the time of the follow-up examination. This was significant for both groups, but greater in absolute terms for patients who underwent additional reduction by the reduction tool. Additionally, patient age at the time of surgery was related on reduction loss. Younger patients showed an overall greater long-term correction than older patients. This could be due to better bone quality and associated better fracture healing at younger ages. Furthermore, preoperative SI/VKA showed a small but not significant influence on the extent of loss. In accordance with previous studies, a greater loss of reduction was observed in patients with worse baseline values [34, 35].

Regarding clinical outcome, no significant difference between the ODI test score of the two groups was found. According to the performed regression analysis, no significant influence of the predictors considered (time between surgery and ODI survey, age at the time of ODI survey, gender, and radiological result at the third measurement time point) on the ODI test result was revealed. However, up to date, almost no studies were able to show an association between posttraumatic kyphosis and impaired clinical outcome [5, 36–38]. Age had the greatest overall influence (beta = 0.28) on the ODI test result, followed by the period between surgery and ODI survey (beta = − 0.23). These findings are in accordance with the literature. A previous study by Muratore et al. showed the same relationship between patient age and ODI test score, whilst Niskanen also observed a relationship between the period of surgery and ODI collection [39, 40]. Factors that influence clinical outcome are diverse and individual [41]. The so far missing or low correlation between radiological result and clinical outcome leads to the assumption that a solitary surgical approach to a complete recovery of the status before the accident may not be sufficient or an almost anatomic reduction may not be necessary.

McCormack et al. reported high implant failure rates after posterior-only stabilization in patients with severe vertebral body defects after suffering incomplete or complete burst fractures [42]. In our study, we did not see any implant failure. We acknowledge that this may be related to a rather short follow-up period, but no implant failure before bony healing was observed. Additionally, 27% of the patients were lost to follow-up. In these patients, hardware failure might have occurred and might been treated at a different facility. Regarding the fact that our department is a referral spine center, patients are routinely sent back for complications.

We acknowledge further limitations of our study. The major limitation of this study was its retrospective design. Therefore, no randomization was performed, and the delay between trauma and surgery is unknown. Additionally, no measurements of the vertebral body height after the initial reduction by positioning were available. In addition to kyphotic sagittal malalignment, coronal plane malalignment may result from vertebral body fractures [6]. This may lead to poor body posture and back pain [43]. This study just focused on kyphosis, and therefore, clinical outcome may also be influenced by coronal vertebral angles. Fracture severity and the grade of instability vary between those fractures. The Nforce reduction tool has no limitation of the applied forces. This may lead also to over-distraction of the anterior cortex and reduced consolidation with consecutive loss of correction. Therefore, a general therapy recommendation cannot be given by this study. It is still necessary to consider each patient individually to choose the most suitable surgical procedure. The lack of long-term follow-up data is another limitation of this study. Additionally, due to the fact that all patients were trauma patients, no preoperative ODI scores were available for comparison. Therefore, further data and randomized controlled studies are warranted.

## Conclusion

According to the results of this study, posterior instrumentation is a suitable method for the initial surgical treatment of complete and incomplete burst fractures of the thoracolumbar junction. Despite significant corrective losses in the postoperative course, overall patient satisfaction was good. The utilization of the reduction tool led to improved correction of the deformity only in young, healthy, and athletic patients. In this collective, additional surgery or vertebral body replacement may be possibly prevented.

## Data Availability

All data are available within the manuscript.
